# miR-140-5p suppresses the proliferation, migration and invasion of gastric cancer by regulating YES1

**DOI:** 10.1186/s12943-017-0708-6

**Published:** 2017-08-17

**Authors:** Zheng Fang, Shuai Yin, Ruochuan Sun, Shangxin Zhang, Min Fu, Youliang Wu, Tao Zhang, Junaid Khaliq, Yongxiang Li

**Affiliations:** 1 Department of General Surgery, First affiliated Hospital of Anhui Medical University, 218 Jixi Avenue, Hefei, 230022 China; 2 Department of General, Visceral, Transplantation, Vascular and Thoracic Surgery, Hospital of University of Munich, Marchioninistr.15, 5H-02-428, 81377 Munich, Germany

**Keywords:** miR-140-5p, gastric cancer, prognostic factor, YES1, tumor progression

## Abstract

**Background:**

The aberrant expression of microRNA-140-5p (miR-140-5p) has been described in gastric cancer (GC). However, the role of miR-140-5p in GC remains unclear. In this study, the prognostic relevance of miR-140-5p in GC was investigated and YES1 was identified as a novel target of miR-140-5p in regulating tumor progression.

**Methods:**

miR-140-5p level was determined in 20 paired frozen specimens through quantitative real-time PCR, and analyzed in tissue microarrays through in situ hybridization. The target of miR-140-5p was verified through a dual luciferase reporter assay, and the effects of miR-140-5p on phenotypic changes in GC cells were investigated in vitro and in vivo.

**Results:**

Compared with that in adjacent normal tissues, miR-140-5p expression decreased in cancerous tissues. The downregulated miR-140-5p in 144 patients with GC was significantly correlated with the reduced overall survival of these patients. miR-140-5p could inhibit GC cell proliferation, migration and invasion by directly targeting 3′–untranlated region of YES1. miR-140-5p could also remarkably reduce the tumor size in GC xenograft mice.

**Conclusions:**

miR-140-5p serves as a potential prognostic factor in patients with GC, and miR-140-5p mediated YES1 inhibition is a novel mechanism behind the suppressive effects of miR-140-5p in GC.

**Electronic supplementary material:**

The online version of this article (doi:10.1186/s12943-017-0708-6) contains supplementary material, which is available to authorized users.

## Background

Gastric cancer (GC) is a frequently diagnosed cancer and considered a major contributor to health problems worldwide [1, 2]. Although diagnostic approaches and treatment strategies have improved gradually, the prognosis of patients with GC in advanced stages remains poor. Thus, the causative mechanisms of GC tumorigenesis and metastasis should be elucidated.

Aberrant signaling events frequently occur in human malignancies and contribute to oncogenesis. For example, upregulated Src signaling, which increases proportionally with progressive TNM stages, has been reported in various human cancers [3], and members of Src family tyrosinekinases (SFKs) are prototypical non-receptor protein tyrosine kinases. YES proto-oncogene 1(YES1), a notable member of SFKs, serves as a seminal regulator of cell growth, adhesion, and differentiation. The major role of YES1 in tumor progression is supported by the decreased YES1 expression correlated with the impaired growth abilities of several malignancies, including malignant mesothelioma, rhabdomyosarcoma, and pancreatic cancer [4–6]. YES1 also provides chemotherapeutic resistance in several cancers. For instance, YES1 can promote the nuclear translocation of the epidermal growth factor receptor that induces resistance to cetuximab in the non-small cell lung cancer line NCI-H226 [7]. However, the expression level of YES1 in GC tissues remains controversial. Seki et al. observed the upregulation of YES1 expression in GC tissues compared with that in adjacent normal tissues, but Wang et al. obtained an opposite conclusion [8, 9]. In addition to these inconsistent findings, the underlying causes of aberrant YES1 expression in GC are unclear.

MicroRNAs (miRNAs) are an endogenous group of small non-coding RNAs that post-transcriptionally repress the expression of protein-coding genes. miRNAs function as specific repressors of translation or cleavage inducers of RNA transcripts by complementarily binding to the corresponding 3′ -untranslated region (3′-UTR) of an mRNA transcribed from a protein-coding gene, miRNAs regulate up to 60% of human protein-coding genes [10], but specific miRNA subsets or clusters are dysregulated in various tumors [11–13]. An example of these miRNAs is miR-140-5p, whose various roles have been widely explored. miR-140-5p has been characterized as a tumor suppressor in colorectal cancer, biliary tract cancer and hypopharyngeal cancer [14–16]. In GC, high miR-140-5p levels have been detected in the plasma of patients, whereas low miR-140-5p levels have been found in 20 tissue samples and a HGC-27 cell line [17, 18]. Nevertheless, the prognostic relevance and functions of miR-140-5p in GC are largely unknown. Considering that various protein coding genes are targeted by one miRNA, researchers should determine the mechanisms of miR-140-5p mediated phenotypic changes in GC cells.

This study aimed to evaluate the prognostic relevance of miR-140-5p in GC and identify whether YES1 is a direct target of miR-140-5p in the regulation of tumor progression.

## Methods

### Patients and tissue samples

Tissue specimens (tumor, adjacent gastric mucosa, and normal tissues from a benign disease) were collected and immediately frozen in liquid nitrogen by a pathologist after informed consent was obtained from patients in the First Affiliated Hospital of Anhui Medical University (Hefei, China). The tissue specimens consisted of 20 paired cancer and adjacent normal tissues from 144 patients with GC and 43 normal gastric mucosa tissues from patients with a benign disease. Histological cancer types were evaluated by at least two independent pathologists in accordance with the TNM staging guide (2010) released by The American Joint Committee on cancer (AJCC). The patients’ characteristics are presented in Additional file 1: Tables S1 and Additional file 2: Table S2. None of the patients received preoperative chemoradiotherapy. Clinical follow-up consisted of telephone calls and reviews of clinical data, including age, gender, tumor location and size. The dates of patients’ death were documented and the overall survival (OS) was defined as the time from the operation date to the date of death. All of the experiments in this study were approved by the Ethics Committee of Anhui Medical University.

### RNA extraction and quantitative real-time PCR (qRT-PCR) analysis

Total RNA was extracted from fresh tissue specimens or cells and assayed for mature miRNAs by using TRIzol reagent (Invitrogen, USA). cDNAs were generated using MMLV reverse transcriptase (Takara, Japan). (qRT-PCR) was run in an IQ5 Real-time PCR detection system (Applied Biosystems, USA), with a SYBR Premix Ex Taq kit (Takara, Japan). The sequences of the primers were listed as follows: miR-140-5p forward primer 5-´TGCGGCAGTGGTTTTACCCTATG- 3′ and reverse primer 5′- CCAGTGCAGGGTCCGAGGT -3′. U6 forward primer 5-´TGCGGGTGCTCGCTTCGGCAGC- 3′ and reverse primer 5′-CCAGTGCAGGGTCCGAGGT -3′. The reactions were incubated at 94 °C for 4 min followed by 40 cycles of 94 °C for 30 s, 58 °C for 30 s and 72 °C for 30 s. Relative expression was achieved by comparative CT calculation (2^-ΔΔCt^ method) and normalized to U6-snRNA. All of the experiments were performed at least in triplicate.

### miRNA In situ hybridization (ISH)

The tissue sections were dewaxed, rehydrated, digested with 15 μg/mL proteinase K, and incubated with a double- DIG- labeled LNA microRNA probe (Exiqon, Denmark) for miR-140-5p with positive U6 (Exiqon, Denmark) and negative scrambled LNA probes as controls. The staining intensities of the tumor cells or normal epithelial cells were scored by two independent pathologists in accordance with the following descriptions: 0, negative, 1, weakly positive, 2 moderately positive and 3 strongly positive [19–21]. Scores ≥2 were designated as “high expression”, whereas scores <2 were defined as “low expression”.

### Cell culture and miRNA transfection

Human cell lines (AGS, MKN45, BGC823, MGC803, SGC7901, GES1 and 293 T) were derived from ATCC (Manassas, Virginia, USA). AGS, MKN45, BGC823, MGC803, and SGC7901 were the five GC cell lines, while GES1 was the immortalized gastric epithelial cell line. AGS, MGC803, GES1 and 293 T cells were cultured in DMEM (Gibco BRL, USA) supplemented with 10% fetal bovine serum (FBS, Gibco BRL, USA), and incubated at 37 °C in a humidified chamber with 5% CO_2_. BGC823, MKN45, and SGC7901 cells were maintained in RPMI 1640 medium (Gibco BRL, USA) with 10% FBS, (Gibco BRL, USA). 293 T cells were transfected with empty pcDNA3.1(+) or constructed pcDNA3.1(+)/YES1 vectors (Saierbo, Tianjin, China). AGS and BGC823 cells were transfected with miR-140-5p or negative control mimics (Gene-Pharma, Shanghai, China) by using Lipofectamine2000 Reagent (Invitrogen, USA) in accordance with the manufacturer’s protocol. Afterward, the GC cells were further transfected with empty pcDNA3.1(+) or pcDNA3.1(+)/YES1 vectors for rescue assays.

### Western blot

GC cells were lysed with RIPA buffer (Beyotime, Jiangsu, China) in an ice bath for 30 min. The lysate was centrifuged at 12,000 rpm for 20 min, and the supernatant was collected. The protein samples in the supernatant were denatured, separated by 10% sodium dodecyl sulfate -polyacrylamide gel-electrophoresis, and transferred onto polyvinylidenedifluoride membranes (Millipore, MA, USA). The membranes were then blocked with 5% skim milk for 1 hand incubated with primary antibodies against YES1, Ki-67, cyclinD1, survivin, E-cadherin, vimentin, Snail, MMP2, MMP3 and SOX4 (1:200, Saierbo, Tianjin, China) at 4 °C overnight. Afterward, the membranes were washed with Tris-buffered saline and incubated with secondary horseradish peroxidase (HRP-) labeled goat anti-rabbit IgG antibodies (1:10,000, Saierbo, Tianjin, China) for 2 h. β-tubulin (1:500.Saierbo, Tianjin, China) was used as control. The protein bands on the membranes were then detected using Western Lightning Chemiluminescence Reagent (PerkinElmer, USA) and photographed through LabWorksGel imaging and analysis system (UVP, USA).

### Dual luciferase reporter assay

HumanYES1 3′ UTR (Accession number NM_014143 for YES1), and mutant YES1 3′ UTR were cloned with the following primers: underlined letters corresponded to the mutated sequences and bold letters indicated the sites cut by PmeI or Xbal).

YES1–3′ UTR wild-type:

- Forward:5′-**AAAC** TAGCGGCCGC TAGT TATGGTTGCACAAAACCACTT **T -**3′, Reverse: 5′-**CTAGA** AAGTGGTTTTGTGCAACCATA ACTA GCGGCCGCTA**GTTT**-3′

YES1–3′ UTR mutant:

- Forward: 5′-**AAAC** TA GCGGCCGC TAGT TATGGTTGCACATATCGAGAT **T-**3′, Reverse: 5′-**CTAGA** ATCTCGATATGTGCAACCATA ACTA GCGGCCGCTA**GTTT**-3′

The fragment of the wild type or mutant YES1 3′ UTR was ligated into a pmirGLO -dual-luciferase miRNA target expression vector (Promega, USA). The constructed vectors were validated by sequencing, cloned, and purified. AGS was then co-transfected with the constructed vectors and miR-140-5p mimics or miR-NC mimics (GenePharma, Shanghai, China) and then cultivated for 48 h. Luciferase activity was subsequently measured using the Dual Luciferase Reporter Gene Assay Kit (Beyotime, Jiangsu, China) in accordance with the manufacturer’s protocol. The relative light unit of the firefly luciferase was recorded with a GloMax 96 microplate luminometer (Promega, USA). The ratio of firefly to Renilla was used to normalize the firefly luciferase values.

### Cell proliferation assay

After transfection was conducted, MTT assay was performed for 24, 48, or 72 h in accordance with the manufacturer’s manual. In brief, 100 μL of a culture medium containing 1 × 10^4^ GCcells were added to each well of a 96well plate. After 12 h of cultivation, 10 μL of MTT solutions were added into each well and incubated for 4 h. Afterward, 100 μL of a detergent reagent was added, and the plate was incubated in the dark at room temperature for 2 h. Absorbance at 490 nm in each well was recorded using a microplate reader (Bio-tek, USA).

### Colony formation assays

GC cells were suspended in DMEM containing 10% FBS and 0.3% low- melting-point agarose. A total of 500 cells were then seeded into each well of a 12-well plate coated with 0.5% agarose. After 14 days of cultivation, colonies were stained with 0.1% crystal violet and counted.

### Wound healing assays

GC cells were suspended and seeded into each well of a12-well plate at a density of 1 × 10^5^/cm^2^. After the cells attached to the bottom and formed a monolayer, linear wounds were scratched with a pipette tip at the center of the monolayer The detached cells were carefully washed off with PBS thrice. Afterward, 1 mL of DMEM (Gibco BRL, USA) containing2%FBS (Gibco BRL, USA) was added into each well, and the wounds were photographed at 0,2,48, and 72 h under a microscope (Olympus, Japan) and the widths of each scratch wound were recorded.

### Transwell assays

Invasion assay was performed in 24-well Transwell chambers (Millipore, USA) in accordance with the manufacturer’s manual. In brief, the invasion chamber was pre-coated with 10 μL of fibronectin(Sciencell, USA) and loaded with 50 μL of Matrigel (BD, USA). After the coated plate was incubated at 37 °C for 2 h, 0.5 mL of cell suspension without serum (1 × 10^5^ cells) was added to the invasion chamber and 500 μL DMEM with 10% FBS, (Gibco BRL, USA) was added to the lower chamber. The plate was then incubated at 37 °C for 24 h, and the cells on the outer surface of the insert bottom were fixed, stained with crystal violet, and counted under a microscope at 100× magnification.

### In vivo xenograft study

A mouse xenograft model was established in 6 week- old BALB/c-nu mice (Model Animal Research Institute of the Chinese Academy of Medical Sciences, Beijing, China) in accordance with the institutional guidelines. In brief, 5 × 10^6^ BGC823 cells suspended in 100 μL of PBS were injected subcutaneously into the left dorsal flanks of the mice. When the tumor size reached approximately 50 mm^3^, 18 mice were equally divided into the following three groups: group A as blank control, group B as miR-140-5p treatment, and group C as control mimic treatment. Each tumor in group B or C was injected with50 μL solution containing **7** nmol miRNA mimics, 3 μL of Lipofectamine2000 (Invitrogen), and 40 μL of serum-free medium Opti-MEM (Invitrogen). After the treatments were administered, the length (L) and width (W) of the tumors were measured every 3 days, and the mice were weighed every 4 days. Tumor volume (V) was calculated using the following eq. V = 0.5 × L × W^2^. On day 19 the mice were sacrificed and the tumors were dissected, weighed and photographed. The tumor tissues were fixed in 4% paraformaldehyde for further studies.

### Immunohistochemistry

The tumor tissues of the mice were fixed, embedded in paraffin. And sliced into consecutive tissue sections. Next, 5 μm thick tissue sections were deparaffinized, dehydrated, and heated in citrate buffer (pH 6.0) for 15 min at 95 °C. To block the nonspecific bindings of the first antibody, we added 1% bovine serum onto the slides for 20 min at room temperature. The tissue sections were then incubated with YES1 antibodies (1:50; Saierbo, Tianjin, China) overnight at 4 °C, and subsequently incubated with the secondary HRP-labeled goat anti-rabbit IgG antibodies (1:20,000, Saierbo, Tianjin, China). Finally, the sections were visualized with a DAB kit (ZSGB-bio, Beijing, China) and counterstained with hematoxylin (Beyotime, Jiangsu, China).

### Statistical analysis

Data were statistically analyzed using GraphpadPrism5. The relative expression of miR-140-5p in the GC tissues and adjacent normal tissues was presented as median (interquartile range) through a nonparametric test (Wilcoxon’s test). Other measurement data were described as means ± standard error of means.

The correlations between miR-140-5p level and the pathologic parameters were analyzed through Pearson’s χ2 test, and the survival and OS curves were plotted using the Kaplan–Meier method. Student’s t test was used to evaluate the differences between two groups, and one-way (ANOVA) was preformed to compare more than two groups. All *P*-values were two-sided and differences were considered significant when *P* < 0.05. *, **, or *** corresponded to *P* < 0.05, *P* < 0.01, or *P* < 0.001, respectively.

## Result

### miR-140-5p is downregulated in GC tissues and correlated with the patients’OS

**Fig. 1:**
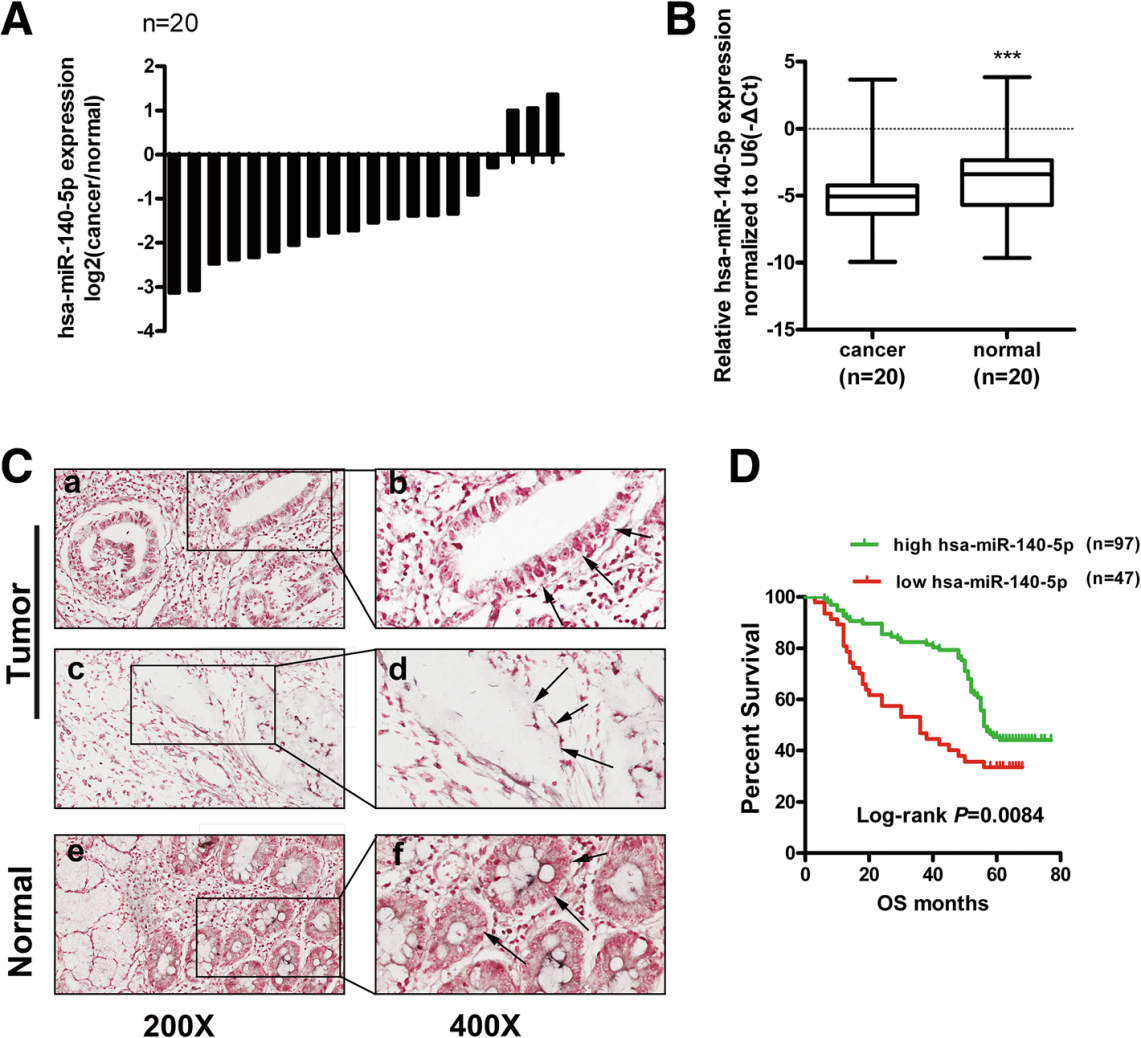
Decreased miR-140-5p expression in tumor tissues correlates with the poor survival of gastric cancer (GC) patients. **a**-**b** miR-140-5p expression significantly decreased in the cancer tissues relative to that in the adjacent normal tissues (**b** presents the medians and interquartile ranges). **c** Representative images of in situ hybridization for miR-140-5p in GC tissues and adjacent normal tissues: *a*, *b* high miR-140-5p level in cancerous epithelia; *c*, *d* low miR-140-5p level in cancerous epithelia; e, f high miR-140-5p level in normal gastric epithelia. The arrows in *b* and *f* orient the strong miR-140-5p staining in cancerous epithelia or normal gastric epithelia, whereas those in d show weak staining in the cancerous epithelia. Magnification:200 × (*a*, *c*, *e*) and 400 × (*b*, *d*, *f*). **d** Overall survival (OS) curves for patients with GC with the log-rank test. Kaplan–Meier survival analysis of the relationship between different miR-140-5p levels and OS of 144 patients with GC

We investigated miR-140-5p expression in 20 fresh GC tissues and matched adjacent normal tissues through qRT-PCR and found that the miR-140-5p expression was significantly downregulated in the tumor tissues (*P* < 0.001) (Fig. 1a-b). Subsequent ISH analysis of the miR-140-5p levels in the 144 GC tissues and 43 normal gastric tissues revealed similar results (Fig. 1c). The staining intensities of miR-140-5p were reduced in 32.6% of the GC tissues (47 of 144).By comparison, 11.6% (5 of 43) of the normal gastric tissues showed downregulations (*P* = 0.007). A Cox proportional hazards regression model was established to examine the prognostic significance of miR-140-5p and other clinical parameters. In addition to tumor differentiation, invasion depth and lymph node metastasis, miR-140-5p level was an independent favorable predictor of OS (Additional file 2: Table S2). Further correlation analysis demonstrated that the downregulated miR-140-5p levels were correlated with the reduced OS of the patients with GC (*P* = 0.0084; Fig. 1d). The median survival times (20 months) of the patients with low miR-140-5p expression were shorter than those (59 months) with high miR-140-5p expression.

### miR-140-5p directly targets one evolutionarily conserved sequence within the 3′UTR of YES1

**Fig. 2:**
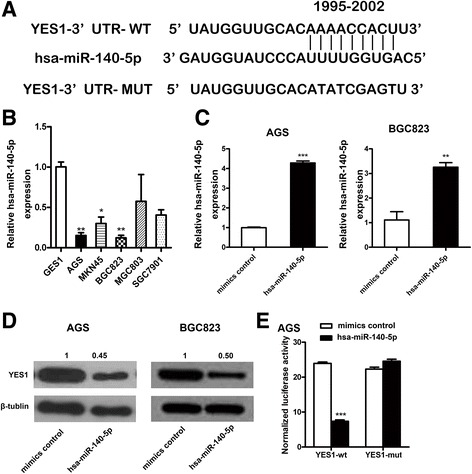
miR-140-5p directly targets YES1 in GC cells. **a** miR-140-5p binding sites of YES1 3′ UTR predicted by the database Targetscan. **b** Expression levels of miR-140-5p determined by quantitative real-time PCR in five GC cell lines and one normal human gastric epithelial cell line GES1. **c** Quantification real-time PCR verification of overexpression after miR-140-5p transfection in AGS and BGC823 cells. **d** Western blot of YES1 protein in GC cells after miR-140-5p or control mimics transfection. **e** Relative luciferase activity in AGS cells co-transfected with miR-140-5p or control mimics together with reporter vectors carrying wild-type (WT) or mutated-type (MT) YES1 3′ UTR

To investigate the potential target of miR-140-5p, we combined the results from prediction databases (TargetScan, miRDB, and ComiR) and found that YES1 was a possible target (Fig. 2a). We then utilized a panel of five GC cell lines and one normal gastric cell line to verify the predicted results. We initially screened the miR-140-5p expression in these cell lines (Fig. 2b) and then confirmed the inverse correlation between miR-140-5p level and YES1 protein expression in AGS and BGC823 cells (*P* < 0.05; Fig. 2c-d). Next, we performed luciferase reporter assay to investigate whether the 3′-UTR of the YES1 gene was a direct target of miR-140-5p in GC. The transient transfection of AGS cells with miR-140-5p and YES1 3′-UTR plasmids revealed that the relative luciferase intensity was significantly decreased compared with that of the miRNA control. By contrast, the vector containing the site mutated 3′-UTR sequence was not affected by miR-140-5p (Fig. 2e). These results indicated that 3′-UTR of YES1 was a direct target of miR-140-5p.

### miR-140-5p suppresses the cell proliferation, migration, and invasion of GC in vitro

**Fig. 3:**
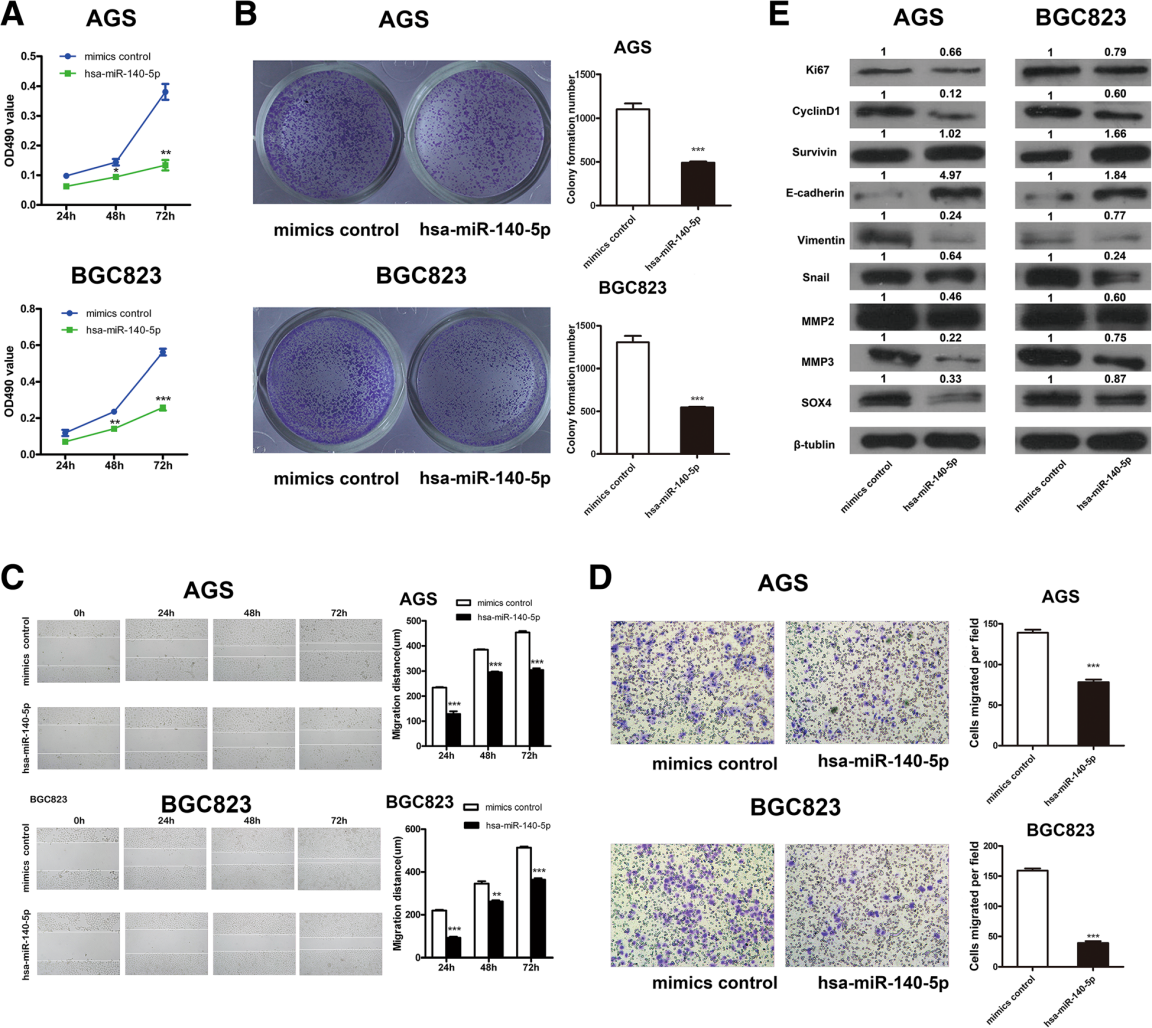
miR-140-5p inhibits GC cell proliferation, migration and invasion in vitro. **a** Effect of miR-140-5p on GC cell growth determined by MTT assays. **b** Colony formation assays in GC cells transfected with miR-140-5p or control mimics. Data are representative of at least three independent experiments; values are expressed as means ± standard errors of the means (SEMs). **c** Wound healing assays showing the inhibition of miR-140-5p on the migratory abilities of GC cells. **d** Transwell assays in GC cells transduced with miR-140-5p or control mimics. Data are representative of at least three independent experiments; values are expressed as means ± SEMs. **e** Western blot analysis of markers associated with tumor proliferation, migration, and invasion, as well as SOX4, after miR-140-5p transfection in vitro

To determine the effects of miR-140-5p on GC cell proliferation, we performed MTT assays and colony formation assays. Compared with those of the control groups in vitro, the transfection-induced overexpression of miR-140-5p in AGS and BGC823 cells significantly suppressed the proliferation ability of GC cells over time (Fig. 3a). The colony formation assays demonstrated that the AGS and BGC823 cells transfected with miR-140-5p showed remarkably decreased colony formation abilities in comparison to those in the control groups (Fig. 3b). Next, we investigated whether miR-140-5p affects GC cell migration and invasion. Wound healing assays showed that the miR-140-5p overexpressing cells were less effective than the non-transfected cells in closing an artificial wound over a confluent monolayer (Fig. 3c). We also examined the influence of miR-140-5p on GC cell invasion by Transwell assay (Fig. 3d). and observed that the numbers of GC cells invading through the Matrigel were remarkably decreased after these cells were treated with miR-140-5p compared with those in the negative control. These results suggested that miR-140-5p attenuated the migration and invasion of GC cells.

### miR-140-5p regulates genes associated with tumor proliferation, migration, and invasion in vitro

Western blot assays were performed to detect the expression of the markers associated with the tumor proliferation, migration, and invasion of AGS and BGC823 cells and to clarify the mechanisms of the inhibitory effects of miR-140-5p on GC cells. After miR-140-5p treatment was administered, the protein levels of Ki-67, CyclinD1, vimentin, Snail, MMP2, and MMP3 were remarkably decreased, whereas the protein level of E-cadherin was significantly increased in both cells. However, the increased level of survivin was observed only in BGC823 cells, whereas no remarkable changes were found in AGS cells. We further verified that the level of SOX4, which has been reported as a direct target of miR-140-5p in HGC-27, was reduced in both cells (Fig. 3e).

### Reconstitution of YES1 rescues the miR-140-5p-mediated inhibition in GC cells

**Fig. 4:**
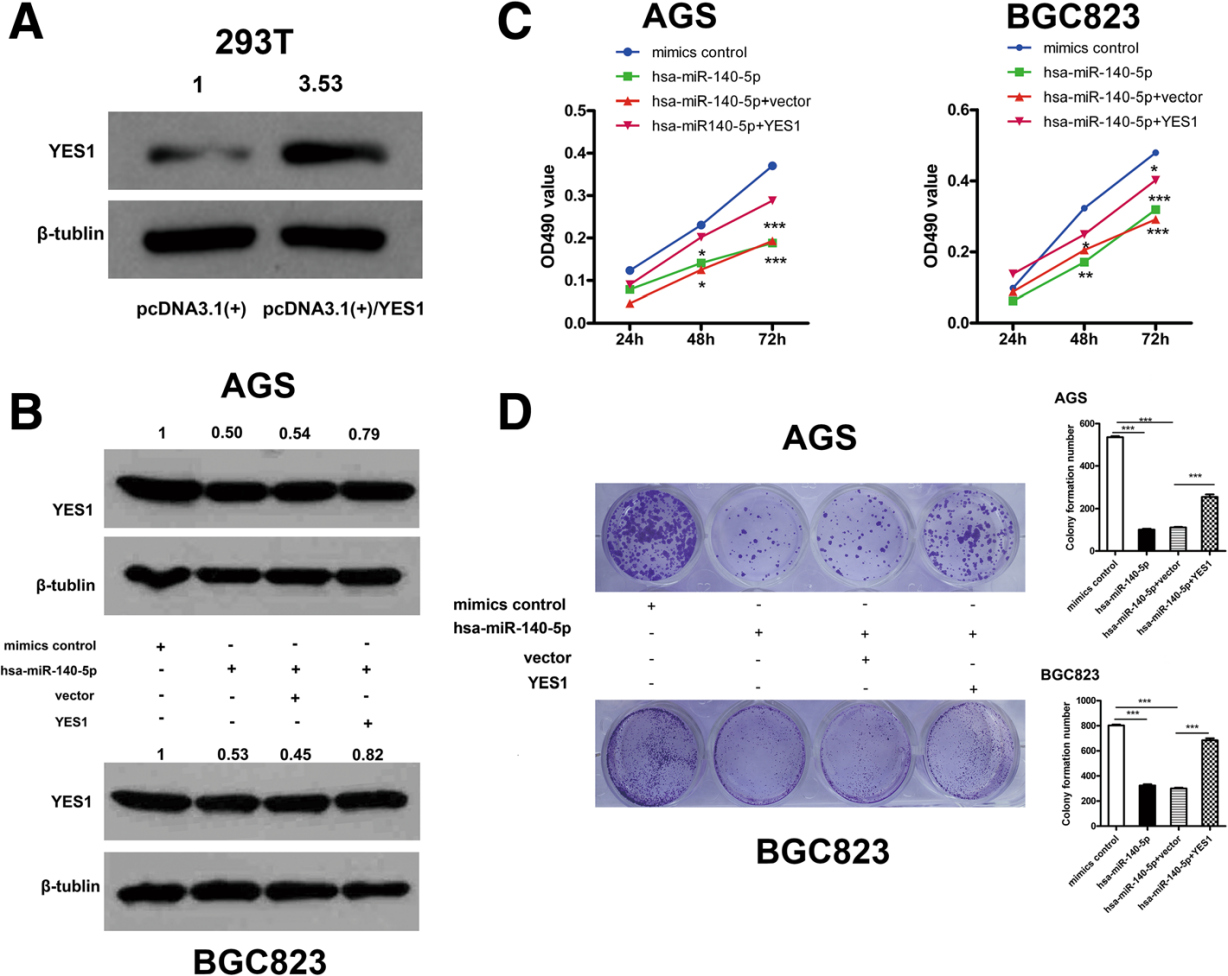
Overexpressing YES1 restores the viability of GC cells in vitro. **a** YES1 protein level of 293 T cells in response to YES1 overexpressing plasmids [pcDNA3.1(+)] or empty vector. **b** Restoration of YES1 expression in GC cells after miR-140-5p treatment as determined by Western blot analysis. **c**-**d** MTT and colony formation assays after overexpressing YES1 in GC cell

**Fig. 5:**
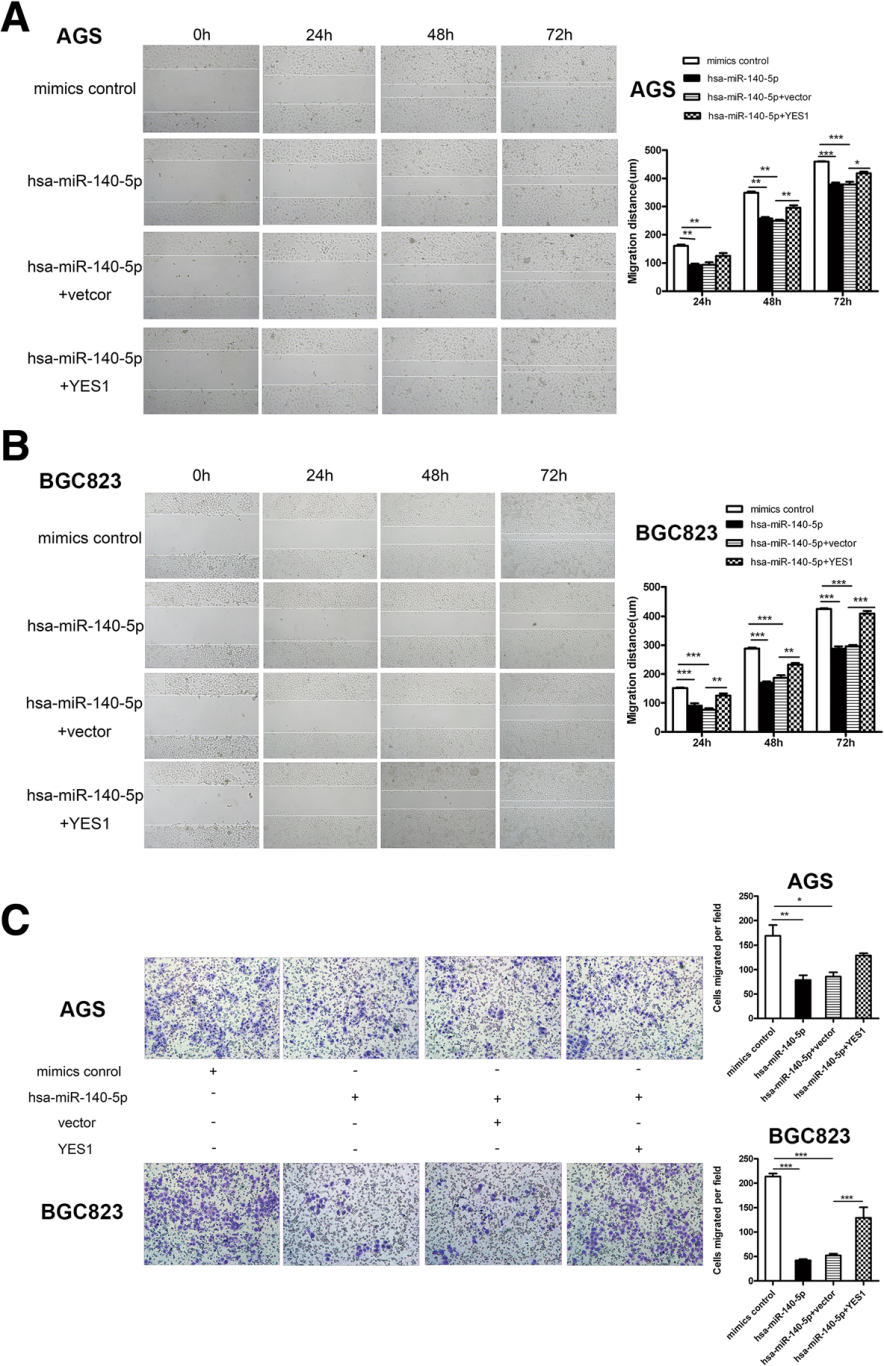
Overexpressing YES1 restores the mobility of GC cells in vitro*.* **a**-**b** Wound healing assays after YES1 re-expression in GC cells. **c** Transwell invasion assays after YES1 reconstitution in GC cells

To examine whether miR-140-5p elicits inhibitory effects on GC cells through YES1, we transfected the constructed YES1 expression vectors with miR-140-5p (Fig. 4a-b). Subsequent MTT and colony forming assay revealed that reintroducing YES1 restored the miR-140-5p-mediated growth repression of GC cells (Fig. 4c-d). Likewise, the repressive effect of miR-140-5p on GC cell migration and invasion could be rescued by reconstituting YES1 (Fig. 5a-c). Collectively, these data provided evidence that miR-140-5p partially repressed the proliferation, migration and invasion of GC cells by inhibiting YES1.

### miR-140-5p suppresses the growth of BGC-823 xenograft tumors

**Fig. 6:**
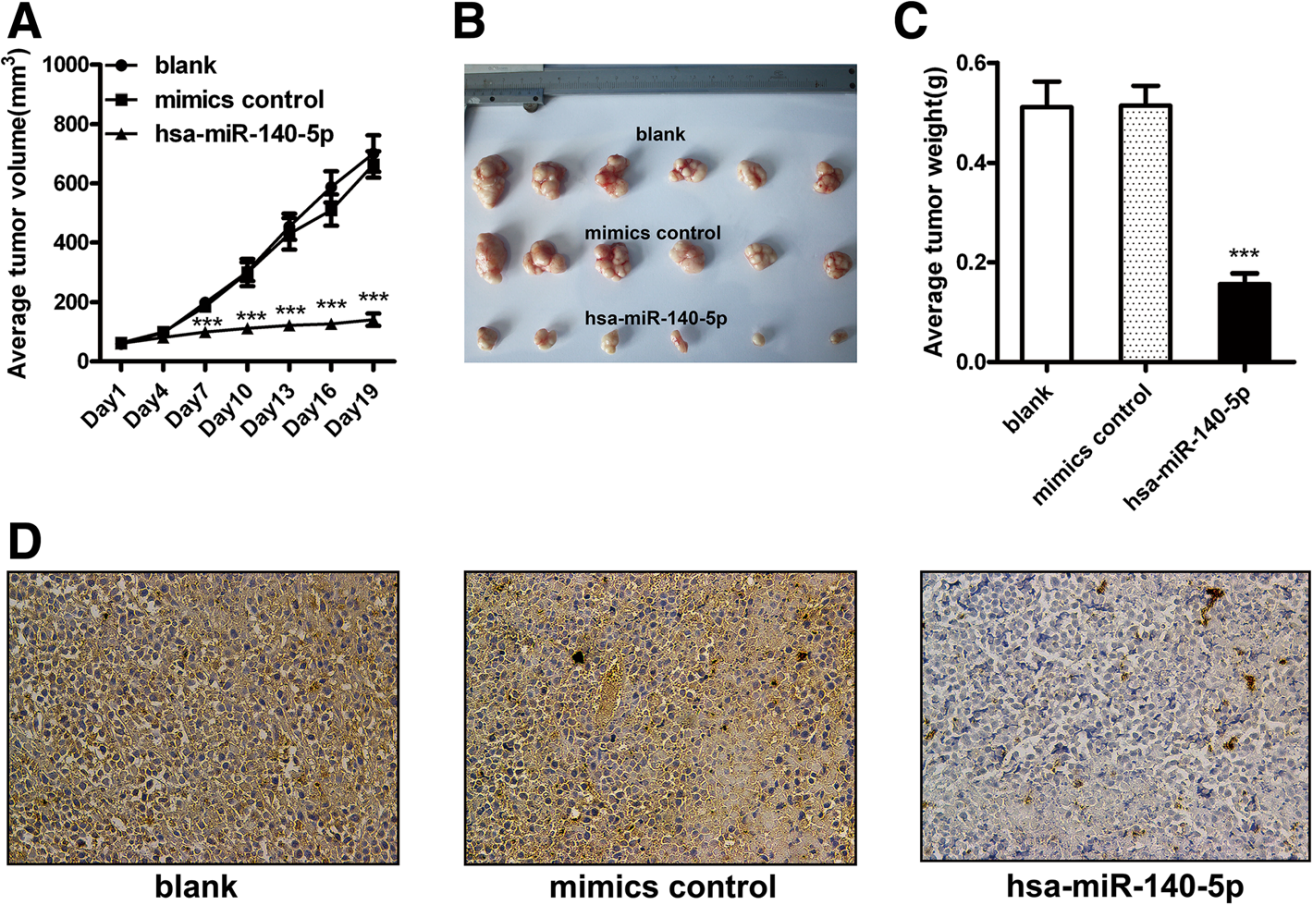
miR-140-5p suppresses the growth of BGC-823 engrafted tumors. **a** Tumor size at different time points after intratumoral injection of miR-140-5p mimics or control mimics. From day7, the tumor volumes were significantly lower in the miR-140-5p groups than in the control groups. **b** Engrafted tumors harvested from the mice. **c** Weights of the xenograft tumors. Data are presented as means ± SEMs. **d** YES1 immunohistochemical staining of the BGC-823 xenograft tumors .Magnification: 200×

On the basis of our in vitro results, we further explored the inhibitory potential of miR-140-5p on GC growth in vivo. BGC-823 cells were subcutaneously implanted into the flank of nude mice and the size of the xenograft tumors was measured after miR-140-5p mimics or control mimics with a blank control were intratumorally injected. The growth curve tended toward lower tumor volumes in the miR-140-5p group than in the control groups (Fig. 6a). Subsequently, the xenograft tumors were harvested and weighed immediately after the mice were sacrificed. Consistent with the tumor volume, the mean tumor mass was significantly lower in the miR-140-5p group than in the control groups (Fig. 6b-c). The YES1 levels in the tumor tissues were than examined through immunohistochemistry staining. In line with the in vitro results, YES1 protein staining was evidently weaker in the miR-140-5p-treated tumors than in the control tumors (Fig. 6d).

## Discussion

Compared with that in adjacent normal tissues, miR-140-5p was downregulated in GC tissues. Similar results were also observed in the GC cell lines compared with those in the nonmalignant cell line. This study is the first to show the correlation between the downregulated expression of miR-140-5p in GC and the reduced mean survival of patients with this disease. We also demonstrated that miR-140-5p directly targeted 3′ UTR-YES1 and the downregulated miR-140-5p was partly responsible for the upregulation of YES1. Moreover, the forced miR-140-5p expression significantly inhibited GC cell proliferation, migration and invasion by inhibiting YES1 in vitro. Intratumoral miR-140-5p injected into the xenograft tumors of the nude mice significantly impeded tumor growth.

A previous study similarly concluded that miR-140-5p levels decrease in GC tissues compared with those in normal tissues. In addition to YES1, other targets, such as SOX4, are downregulated by increased miR-140-5p in HGC-27 [18]. However, these data were obtained from 20 GC samples and 1 GC cancer cell line. Studies with large sample sizes are required to further corroborate these findings. In our study, the ISH staining results of TMA from the144 patients are consistent with the conclusions of other groups. We demonstrated that low miR-140-5p levels in GC tissues predicted the poor clinical outcomes of patients, and we suggested that miR-140-5p could be a potential prognostic factor. Unfortunately, we did not conserve the blood sample of the patients enrolled in the TMA study and thus failed to verify the previous description of serum miR-140-5p levels in patients with GC. To achieve translational application, we will further investigate the correlation of serum miR-140-5p levels with patient prognosis.

Previous studies suggested the diversity of miR-140-5p mediated effects on tumor cell phenotypes. For instance, miR-140-5p suppresses the self-renewal and growth of breast cancer stem cells [22] and inhibits the invasion of esophageal cancer cells rather than their proliferation [23]. However, studies have yet to clarify whether miR-140-5p can induce phenotypic changes, in addition to its inhibitory effects on tumor cell proliferation, in GC. Cancer cell lines might also respond differently to the same treatment because of their genetic variances. As such, additional functional experiments are necessary to support previous conclusions. In the two cell lines observed in our study, miR-140-5p inhibited not only the proliferation of GC cells but also their migration and invasion. These results were further validated by the reduced levels of phenotype-related proteins, including Ki-67 and cyclinD1 (proliferation) [24], vimentin and Snail (migration) [25], and MMP2 and MMP3 (cell invasion) [26, 27]. E-cadherin levels were also increased, and this finding implied that the cell-to-cell adhesion abilities of GC cells are enhanced [28]. Considering the same phenotypic changes of both cell lines, the inconsistent changes of survivin (cell division) levels were possibly due to the different cell line backgrounds; this topic requires further research. Moreover, the upregulation of survivin in BGC823 cells, which indicates the increased abilities of cell division, seemed to be contrary to the growth inhibition phenomenon after miR-140-5p treatment [29]. Thus, we speculate that cell division process might not be the key procedures interfered by miR-140-5p. Additionally, in vitro assays may be unable to show the actual biological processes of tumor cells in complex microenvironments. Thus, we detected the suppressive effect of miR-140-5p by using a nude mouse model. Consistent with our in vitro findings, our observations in the mouse model revealed that miR-140-5p impeded the growth of GC tumor grafted in nude mice.

YES1 is suppressed by miRNAs in the tumor progression of oral cancer, ovarian cancer, and hepatocellular carcinoma, and this observation indicates that YES1 may be regulated by miRNAs in GC [30–32]. Although in silico analysis suggests that YES1 is a target gene of miR-140-5p, neither the putative interaction nor the functional role of YES1 in GC has been defined. Our results show that miR-140-5p directly targeted the 3′-UTR of the YES1 gene, because its overexpression was significantly associated with luciferase activity suppression. A significant decrease in YES1 protein expression was validated after miR-140-5p was overexpressed in GC cells. Likewise, miR-140-5p injections reduced the immunohistochemical staining density of theYES1 protein in the transplanted tumors of nude mice.

Considering the importance of YES1 in solid cancer, we further characterized its role by overexpressing the YES1 protein. YES1, which is a SFK family member, plays a pleiotropic role in oncogenic cell functions. Rosenbluh et al. .confirmed that YES1 promotes the phosphorylation of YAP1 and the proliferation of cancer cells by activating the Wnt/β-catenin pathway [33]. YES1 upregulation similarly reverses the miR-140-5p-mediated inhibition of GC cell growth and colony formation. Liu et al. reported that activating YES1 enhances the metastatic behaviors of melanoma cells [34]. Overexpressing YES1 reverses the inhibitory effects of miR-140-5p on GC cell migration and invasion. We also demonstrated the role of YES1 in the multiple oncogenic activities of GC cells. Interestingly, the co-transfection of YES1-overexpressing vectors with miR-140-5p did not fully revert the suppressive behavior of GC induced by miR-140-5p. This phenomenon could be possibly attributed to the ability of miR-140-5p to directly target other important oncogenic genes, such as SOX4. Previous studies confirmed that miR-140-5p suppresses GC cell proliferation by targeting SOX4, and this finding was supported by our results, which demonstrated that miR-140-5p overexpression decreased the protein levels of SOX4 in AGS and BGC823 cells. Unfortunately, SOX4 has been reportedly associated with the proliferation phenotype of HGC-27 only, and this observation may not explain why YES1 only partially restores the migratory and invasive phenotypes suppressed by miR-140-5p. We inferred that other published miR-140 targets, such as ADAMTS5 and IGFBP5 in colorectal cancer [35], Pin1 in liver cancer [36], and IGF2BP1 in cervical cancer [37], which are associated with tumor migration and invasion, may contribute to these GC cell phenotypes. However, further studies should identify the novel pathways by which miR-140-5p mediates its inhibitory effects on GC because of the genetic diversities of different tumors, and the genetic variances of different cell lines from the same tumor type.

## Conclusions

This study is the first to report that miR-140-5p serves as a potential prognostic factor for patients with GC and inhibits GC tumorigenesis by directly targeting YES1. Our study provides further insights into the pathogenesis of GC and basis for improvements of therapeutic approaches.

## Additional files

Additional file 1: Table S1. Correlations between the miR-140-5p levels and the clinicopathologic features of the 144 patients with GC. (XLS 23 kb)

Additional file 2: Table S2. Relationship between the clinicopathologic parameters and the overall survival of the patients with GC. (XLS 21 kb)

## Abbreviations

3’UTR: 3′ untranslated region
GC: Gastric cancer
ISH: in situ hybridization
qRT-PCR: quantitative real-time polymerase chain reaction
SFKs: Src family tyrosine kinase
YES1: YES proto-oncogene 1

## References

[1] Siegel R, Ma J, Zou Z, Jemal A (2014). Cancer statistics, 2014. CA Cancer J Clin.

[2] Chen W, Zheng R, Zeng H, Zhang S (2016). The incidence and mortality of major cancers in China, 2012. Chin J Cancer.

[3] Leroy C, Shen Q, Strande V, Meyer R, McLaughlin ME, Lezan E, Bentires-Alj M, Voshol H, Bonenfant D, Alex Gaither L (2015). CUB-domain-containing protein 1 overexpression in solid cancers promotes cancer cell growth by activating Src family kinases. Oncogene.

[4] Sato A, Sekine M, Virgona N, Ota M, Yano T (2012). Yes is a central mediator of cell growth in malignant mesothelioma cells. Oncol Rep.

[5] Yeung CL, Ngo VN, Grohar PJ, Arnaldez FI, Asante A, Wan X, Khan J, Hewitt SM, Khanna C, Staudt LM, Helman LJ (2013). Loss-of-function screen in rhabdomyosarcoma identifies CRKL-YES as a critical signal for tumor growth. Oncogene.

[6] Je DW, O YM, Ji YG, Cho Y, Lee DH (2014). The inhibition of SRC family kinase suppresses pancreatic cancer cell proliferation, migration, and invasion. Pancreas.

[7] Iida M, Brand TM, Campbell DA, Li C, Wheeler DL (2013). Yes and Lyn play a role in nuclear translocation of the epidermal growth factor receptor. Oncogene.

[8] Seki T, Fujii G, Mori S, Tamaoki N, Shibuya M (1985). Amplification of c-yes-1 proto-oncogene in a primary human gastric cancer. Jpn J Cancer Res.

[9] Wang L, Zhu JS, Song MQ, Chen GQ, Chen JL (2006). Comparison of gene expression profiles between primary tumor and metastatic lesions in gastric cancer patients using laser microdissection and cDNA microarray. World J Gastroenterol.

[10] Friedman RC, Farh KK, Burge CB, Bartel DP (2009). Most mammalian mRNAs are conserved targets of microRNAs. Genome Res.

[11] Murphy BL, Obad S, Bihannic L, Ayrault O, Zindy F, Kauppinen S, Roussel MF (2013). Silencing of the miR-17~92 cluster family inhibits medulloblastoma progression. Cancer Res.

[12] Li Y, Li W, Ying Z, Tian H, Zhu X, Li J, Li M (2014). Metastatic heterogeneity of breast cancer cells is associated with expression of a heterogeneous TGFbeta-activating miR424-503 gene cluster. Cancer Res.

[13] Chen S, Sun KX, Liu BL, Zong ZH, Zhao Y (2016). MicroRNA-505 functions as a tumor suppressor in endometrial cancer by targeting TGF-alpha. Mol Cancer.

[14] Zhang W, Zou C, Pan L, Xu Y, Qi W, Ma G, Hou Y, Jiang P (2015). MicroRNA-140-5p inhibits the progression of colorectal cancer by targeting VEGFA. Cell Physiol Biochem.

[15] Yu J, Zhang W, Tang H, Qian H, Yang J, Zhu Z, Ren P, Lu B (2016). Septin 2 accelerates the progression of biliary tract cancer and is negatively regulated by mir-140-5p. Gene.

[16] Jing P, Sa N, Liu X, Liu X, Xu W (2016). MicroR-140-5p suppresses tumor cell migration and invasion by targeting ADAM10-mediated Notch1 signaling pathway in hypopharyngeal squamous cell carcinoma. Exp Mol Pathol.

[17] Shin VY, Ng EK, Chan VW, Kwong A, Chu KM (2015). A three-miRNA signature as promising non-invasive diagnostic marker for gastric cancer. Mol Cancer.

[18] Zou J, Xu Y (2016). MicroRNA-140 inhibits cell proliferation in gastric cancer cell line HGC-27 by suppressing SOX4. Med Sci Monit.

[19] Donnem T, Eklo K, Berg T, Sorbye SW, Lonvik K, Al-Saad S, Al-Shibli K, Andersen S, Stenvold H, Bremnes RM, Busund LT (2011). Prognostic impact of MiR-155 in non-small cell lung cancer evaluated by in situ hybridization. J Transl Med.

[20] Donahue TR, Nguyen AH, Moughan J, Li L, Tatishchev S, Toste P, Farrell JJ (2014). Stromal microRNA-21 levels predict response to 5-fluorouracil in patients with pancreatic cancer. J Surg Oncol.

[21] Qadir XV, Han C, Lu D, Zhang J, Wu T (2014). miR-185 inhibits hepatocellular carcinoma growth by targeting the DNMT1/PTEN/Akt pathway. Am J Pathol.

[22] Li Q, Yao Y, Eades G, Liu Z, Zhang Y, Zhou Q (2014). Downregulation of miR-140 promotes cancer stem cell formation in basal-like early stage breast cancer. Oncogene.

[23] Li W, Jiang G, Zhou J, Wang H, Gong Z, Zhang Z, Min K, Zhu H, Tan Y (2014). Down-regulation of miR-140 induces EMT and promotes invasion by targeting Slug in esophageal cancer. Cell Physiol Biochem.

[24] Manu KA, Shanmugam MK, Ramachandran L, Li F, Fong CW, Kumar AP, Tan P, Sethi G (2012). First evidence that gamma-tocotrienol inhibits the growth of human gastric cancer and chemosensitizes it to capecitabine in a xenograft mouse model through the modulation of NF-kappaB pathway. Clin Cancer Res.

[25] Zhao L, Li W, Zang W, Liu Z, Xu X, Yu H, Yang Q, Jia J (2013). JMJD2B promotes epithelial-mesenchymal transition by cooperating with beta-catenin and enhances gastric cancer metastasis. Clin Cancer Res.

[26] Bae IH, Park MJ, Yoon SH, Kang SW, Lee SS, Choi KM, Um HD (2006). Bcl-w promotes gastric cancer cell invasion by inducing matrix metalloproteinase-2 expression via phosphoinositide 3-kinase, Akt, and Sp1. Cancer Res.

[27] Shoshan E, Braeuer RR, Kamiya T, Mobley AK, Huang L, Vasquez ME, Velazquez-Torres G, Chakravarti N, Ivan C, Prieto V (2016). NFAT1 directly regulates IL8 and MMP3 to promote melanoma tumor growth and metastasis. Cancer Res.

[28] Shi Z, Zhang J, Qian X, Han L, Zhang K, Chen L, Liu J, Ren Y, Yang M, Zhang A (2013). AC1MMYR2, an inhibitor of dicer-mediated biogenesis of Oncomir miR-21, reverses epithelial-mesenchymal transition and suppresses tumor growth and progression. Cancer Res.

[29] Connell CM, Wheatley SP, McNeish IA (2008). Nuclear survivin abrogates multiple cell cycle checkpoints and enhances viral oncolysis. Cancer Res.

[30] Lee SA, Kim JS, Park SY, Kim HJ, Yu SK, Kim CS, Chun HS, Kim J, Park JT, Go D, Kim do K (2015). miR-203 downregulates Yes-1 and suppresses oncogenic activity in human oral cancer cells. J Biosci Bioeng.

[31] Li L, He L, Zhao JL, Xiao J, Liu M, Li X, Tang H (2015). MiR-17-5p up-regulates YES1 to modulate the cell cycle progression and apoptosis in ovarian cancer cell lines. J Cell Biochem.

[32] Tan W, Lim SG, Tan TM (2015). Up-regulation of microRNA-210 inhibits proliferation of hepatocellular carcinoma cells by targeting YES1. World J Gastroenterol.

[33] Rosenbluh J, Nijhawan D, Cox AG, Li X, Neal JT, Schafer EJ, Zack TI, Wang X, Tsherniak A, Schinzel AC (2012). beta-Catenin-driven cancers require a YAP1 transcriptional complex for survival and tumorigenesis. Cell.

[34] Liu W, Monahan KB, Pfefferle AD, Shimamura T, Sorrentino J, Chan KT, Roadcap DW, Ollila DW, Thomas NE, Castrillon DH (2012). LKB1/STK11 inactivation leads to expansion of a prometastatic tumor subpopulation in melanoma. Cancer Cell.

[35] Yu L, Lu Y, Han X, Zhao W, Li J, Mao J, Wang B, Shen J, Fan S, Wang L (2016). microRNA −140-5p inhibits colorectal cancer invasion and metastasis by targeting ADAMTS5 and IGFBP5. Stem Cell Res Ther.

[36] Yan X, Zhu Z, Xu S, Yang LN, Liao XH, Zheng M, Yang D, Wang J, Chen D, Wang L (2017). MicroRNA-140-5p inhibits hepatocellular carcinoma by directly targeting the unique isomerase Pin1 to block multiple cancer-driving pathways. Sci Rep.

[37] Su Y, Xiong J, Hu J, Wei X, Zhang X, Rao L. MicroRNA-140-5p targets insulin like growth factor 2 mRNA binding protein 1 (IGF2BP1) to suppress cervical cancer growth and metastasis. Oncotarget. 2016;7:68397–411. 10.18632/oncotarget.11722 PMC5356564 27588393

